# Selection of 1-mm venting or 2.5-mm screw access holes on implant crowns based on cement extrusion and retention capacity

**DOI:** 10.1186/s12903-022-02145-x

**Published:** 2022-04-02

**Authors:** Huangjun Zhou, Sixian Ye, Min Liu, Hao Feng, Cai Wen

**Affiliations:** 1 Department of Oral Implantology, The Affiliated Stomatological Hospital of Southwest Medical University, Luzhou, Sichuan China; 2 Luzhou Key Laboratory of Oral and Maxillofacial Reconstruction and Regeneration, The Affiliated Stomatological Hospital of Southwest Medical University, Luzhou, Sichuan China; 3 Department of Prosthodontics, The Affiliated Stomatological Hospital of Southwest Medical University, Luzhou, Sichuan China; 4 Department of Oral and Maxillofacial Surgery, The Affiliated Stomatological Hospital of Southwest Medical University, Luzhou, Sichuan China; 5 Department of VIP Dental Service, The Affiliated Stomatological Hospital of Southwest Medical University, Luzhou, Sichuan China

**Keywords:** Implant crown, Screw access hole, Cement-retained, Excess cement, Retentive strength, Hole diameter

## Abstract

**Background:**

This in vitro study aimed to provide evidence regarding the selection of hole diameters of implant crowns to reduce excess cement extrusion at the abutment margin, and to examine the maintenance of their retention capacity in anterior and posterior cement-retained implant crowns.

**Methods:**

Six groups of implant crowns were prepared according to the position of the teeth and the size of their holes as follows: anterior crown without hole (ANH), anterior crown with 1-mm mini venting hole (AMH), anterior crown with 2.5-mm regular screw access hole (ARH), posterior crown without hole (PNH), posterior crown with 1-mm mini venting hole (PMH), and posterior crown with 2.5-mm regular screw access hole (PRH). Temporary cement was used to bond the crowns to the abutments. The mean amount of excess cement extrusion among the different groups at the abutment margin was calculated. Retentive strength under different hole designs was measured as the dislocation force of the crown using a universal testing machine. One-way ANOVA and Welch’s t-test were used to analyze the results.

**Results:**

The average amounts of extruded excess cement were 18.96 ± 0.64, 1.78 ± 0.41, and 1.30 ± 0.41 mg in the ANH, AMH, and ARH groups, respectively, and 14.87 ± 0.36, 1.51 ± 0.40, and 0.82 ± 0.22 mg in the PNH, PMH, and PRH groups, respectively. The hole opening in the crowns could significantly reduce residual cement regardless of its size (p < 0.001). The mean retentive strengths were 54.16 ± 6.00, 47.63 ± 13.54, and 31.99 ± 7.75 N in the ANH, AMH, and ARH groups, respectively, and 57.84 ± 10.19, 53.22 ± 6.98, and 39.48 ± 5.12 N in the PNH, PMH, and PRH groups, respectively. The retention capacity of the implant crown deteriorated rapidly as the holes on the crown surface enlarged.

**Conclusions:**

The presence of a hole on the implant crown reduced the amount of excess cement. The retention ability of the implant crowns deteriorated as the size of the hole increased. Considering the esthetic effect of the crown and the possible influence on crown retention, an implant crown with a 1-mm mini venting hole is a better clinical choice than the one with a 2.5-mm regular screw access hole.

## Background

Since the five-year success rate of dental implants has been above 90%, partially and completely edentulous patients tend to choose implant-supported prostheses to restore their teeth [1–6]. The retention methods of implant-supported restoration are mainly of two types: screw-retained and cement-retained [7]; both have advantages and disadvantages. There is no cement residue in screw-retained restoration; however, the screws could loosen or fracture. In contrast, for cement-retained implant restoration, it is easier to achieve passive fit [8, 9], and there is a lower risk of screw loosening or fracture, fewer structural components, and lower prosthodontic cost. Cement-retained implant crowns are popular among prosthodontists in clinical practice because of its convenience; however, residual excess cement is difficult to avoid.

Residual excess cement is a common complication of cement-retained prostheses. It is difficult to remove and may lead to a high risk of peri-implant diseases [10–12]. According to a review conducted by Staubli et al. [13], the prevalence of peri-implant diseases in cement-retained implant restorations ranges from 2–75%, and 33–100% of those cases are related to excess cement extrusion at the abutment margin. Based on the operation procedure of cementation, complete avoidance of cement residue extrusion seems inevitable, and the amount of cement residue increases as the margin of restoration is placed deeper [14–16]. The amount of cement residue required to cause peri-implant diseases has not yet been determined; however, it is advantageous to minimize cement residue [17, 18]. Some experts suggest that creating a hole in the crown could be a simple and convenient way to reduce the cement extrusion at the margin during cementation, and that the hole can also serve as a marker of abutment access when retrieving the crown [19–21].

Implant crowns with venting or access holes have several advantages; however, the hole on the crown surface may aggravate the integrity and esthetics of the crown and affect its retention capacity and fracture resistance strength. The necessity of hole opening and its optimization have not been discussed in depth, and there has been no consensus on the designs for hole size. In clinical practice, the personal preference of the dentist affects whether hole opening is applied as well as the size of the hole for the implant crown.

Therefore, this in vitro study aimed to evaluate the effect of hole diameters on the reduction of excess cement extrusion and retentive strength of implant crowns. The null hypothesis was that the size of the hole on the implant crown significantly affects the amount of cement extrusion at the abutment margin during cementing and retention strength after bonding.

## Methods

### Specimen preparation

Since this study was conducted solely in vitro, ethical approval was not required. Fabrication of anterior and posterior implant crowns included 9 titanium abutments (4.5 mm diameter and 2.5 mm G/H, Dentium, South Korea) and 9 abutments (5.5 mm diameter and 2.5 mm G/H, Dentium, South Korea) which were attached to implant analogs with a torque of 30 N cm; high-strength resin material was used to fabricate implant-supported crowns. The anterior and posterior crowns were fabricated according to the morphology of the maxillary central incisor and the mandibular first molar, respectively. Hooks were fabricated on both sides of the crowns to facilitate the retention strength tensile test.

**Fig. 1:**
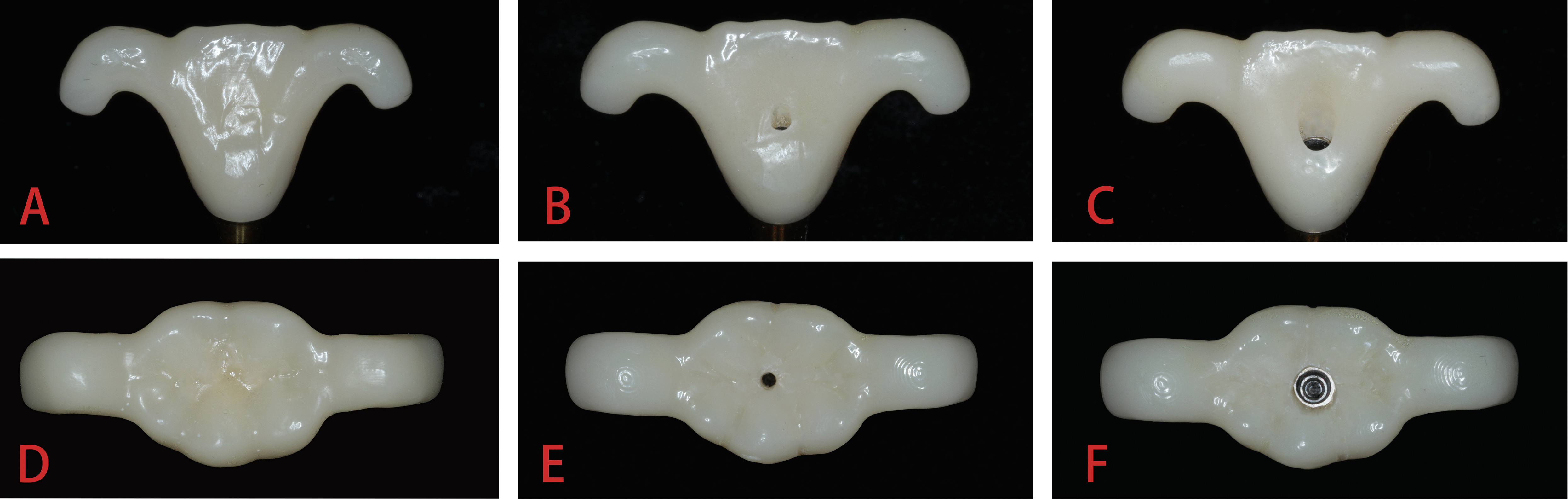
Implant crowns with venting and screw access holes of different diameters. **A**, **B**, **C**: anterior crowns; **D**, **E**, **F** posterior crowns. Specimens from left to right: crowns with no hole (NH), crowns with a 1-mm mini venting hole (MH), and crowns with a 2.5-mm regular screw access hole (RH)

According to the tooth position and diameter of the holes on the crowns, the groups were set as follows: Group A, anterior crowns with no hole (ANH); Group B, anterior crowns with a 1-mm mini venting hole (AMH); Group C, anterior crowns with a 2.5-mm regular screw access hole (ARH); Group D, posterior crowns with no hole (PNH); Group E, posterior crowns with a 1-mm mini venting hole (PMH); and Group F, posterior crowns with a 2.5-mm regular screw access hole (PRH). These six groups are illustrated in Fig. 1; each group included 9 specimens. The abutment screw access channels were filled with light-curing onlay resin (Systemp Onlay, Ivoclar Vivadent, Liechtenstein), and the height of the resin filling was flush with the abutment edge.

The holes on the crown surface were opened at the corresponding position of the abutment screw access channel, which was labial to the lingual fossa in the maxillary central incisor and to the central fossa in the mandibular first molar. The holes on the crowns were reamed step-by-step, and the cementing and retentive strength experiments were repeated on the same crown with different hole sizes to eliminate the influence of irrelevant factors, such as differences in the inner surface morphology and the volume of the gap between the crown and the abutment between specimens. The experiment started with no-hole (NH) groups (Groups A and D), then1-mm mini venting hole (MH) groups (Groups B and E), and finally 2.5-mm regular screw access hole (RH) groups (Groups C and F). The diameter of the hole was increased in sequence using a high-speed handpiece with tungsten carbide fissure burs (1-mm). A standardized practice for hole opening and water cooling were ensured and conducted by the same experimenter.

Prior to initiating the next experiment, the residual cement inside the crowns was carefully cleaned: solidified cement in bulk was removed using a probe, and cement fragments were cleaned using a cotton swab with 95% alcohol. Subsequently, the crowns were treated with an ultrasonic cleaning machine (Shumei KQ5200E, Kun Shan Ultrasonic instruments, China) for 30 min to completely remove the cement residue and dried with oil-free air.

### Cementation experiments

Temporary cement (Tempbond NE, Kerr, USA) was used for the cementing experiment. A clean plastic spatula was used to mix the base and accelerator of the cement according to the manufacturer's instructions. The loading amount of cement was standardized to approximately 30 mg each time, which was determined from a pilot study. The experimenter used a probe to coat the cement evenly on the internal walls of the crown, and the crown was fully and passively seated on the abutment with the maximum finger pressure for over 1 min until the cement was completely cured.

**Fig. 2:**
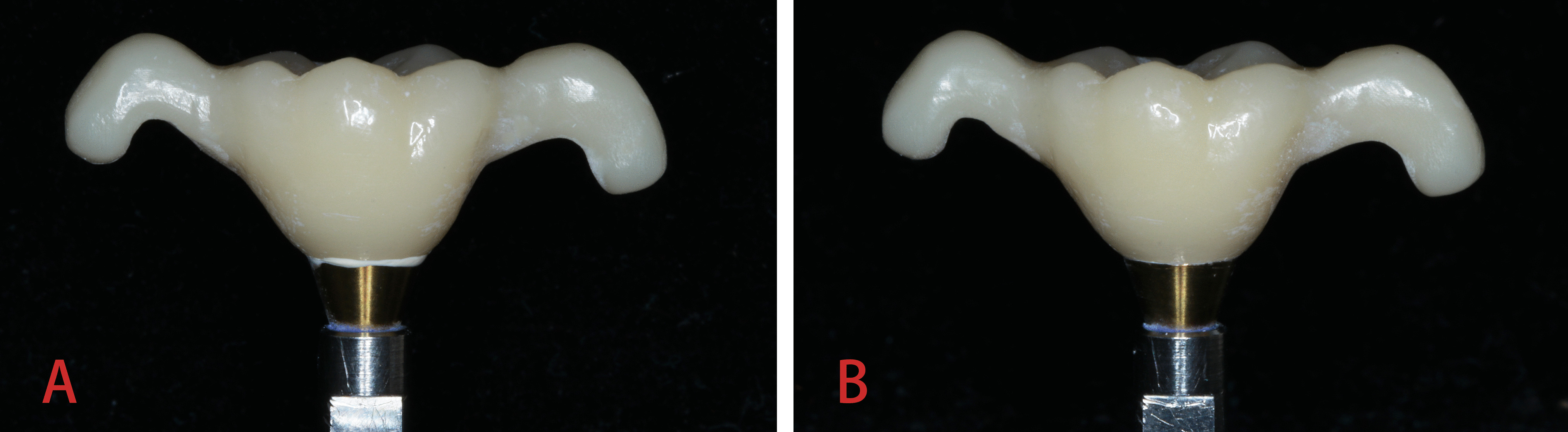
Images depicting the abutment margin of the crown. **A** Excess cement extruded at the abutment margin of the crown. **B** After careful removal of excess cement at the abutment margin of the crown

The specimen was then weighed on an electronic scale (Mettler LE204E, Mettler Toledo, China) with an accuracy of 0.0001 g, and its weight was recorded as the pre-cement-removal weight (W1 [mg]). The excess cement extruded at the margin of the abutment was carefully removed using a probe and a scraper, and the specimen was scrubbed with cotton (Fig. 2). The specimen was weighed on the electronic scale again, and the post-cement-removal weight (W2 [mg]) was recorded. The weight of the extruded excess cement at the abutment margin was calculated using the following formula: ΔW(mg) = W1-W2. The weight measurements were repeated three consecutive times for a single specimen to obtain the mean score.

### Retentive strength test

**Fig. 3:**
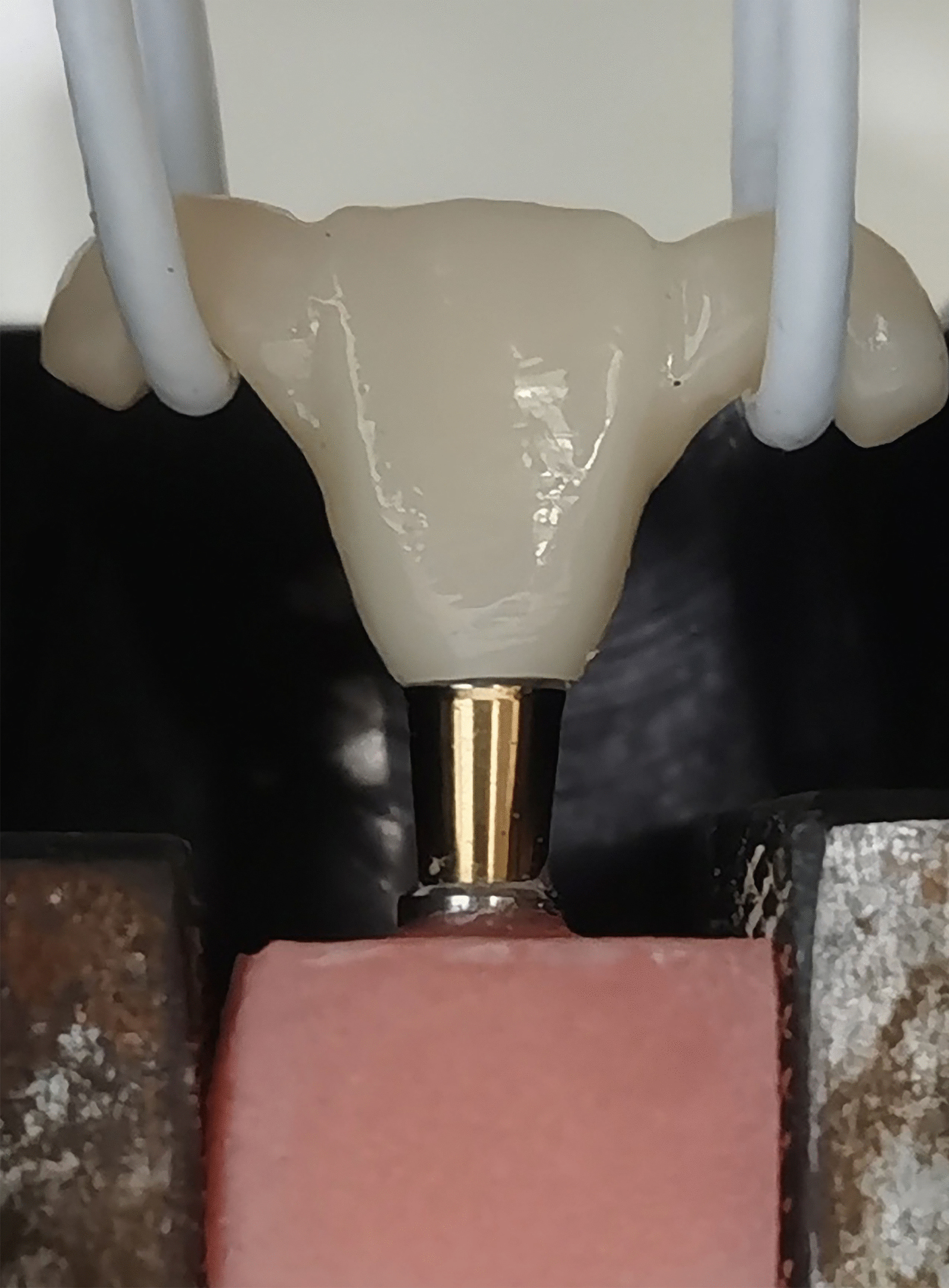
Retentive strength test for anterior crown specimens, conducted by a universal testing machine

The cemented specimens were placed in distilled water and stored at 37 °C for 24 h. The retentive strength of the specimen was evaluated by the tensile force required to separate the crown from the abutment. The hooks on both sides of each crown were attached to a universal testing machine (WDW-20, JINAN YNSJ, China), and the crown-abutment unit was fixed on the testing machine by polymethyl methacrylate (Fig. 3). The tensile force was applied parallel to the long axis of the specimens at a crosshead speed of 1 mm/min until the crown dislodged from the abutment; load–deflection curves were used to record the tensile force, and the tensile force applied to separate the specimen was obtained from the curve and recorded in Newtons (N).

### Statistical analysis

Statistical analyses were performed using Statistical Package for the Social Sciences software (SPSS 19.0, IBM, USA). Levene's test for homogeneity of variance was conducted. One-way ANOVA was used to analyze the differences in the amount of cement extruded at the abutment margin as well as the retentive strength of crowns with varying hole diameters if homogeneity of variance held true (p > 0.05); otherwise, Welch’s ANOVA was used. Pairwise comparisons for subgroups were analyzed using the Student–Newman–Keuls or Games-Howell test. Statistical significance was set at p < 0.05.

## Results

### Excess cement extrusion

**Fig. 4:**
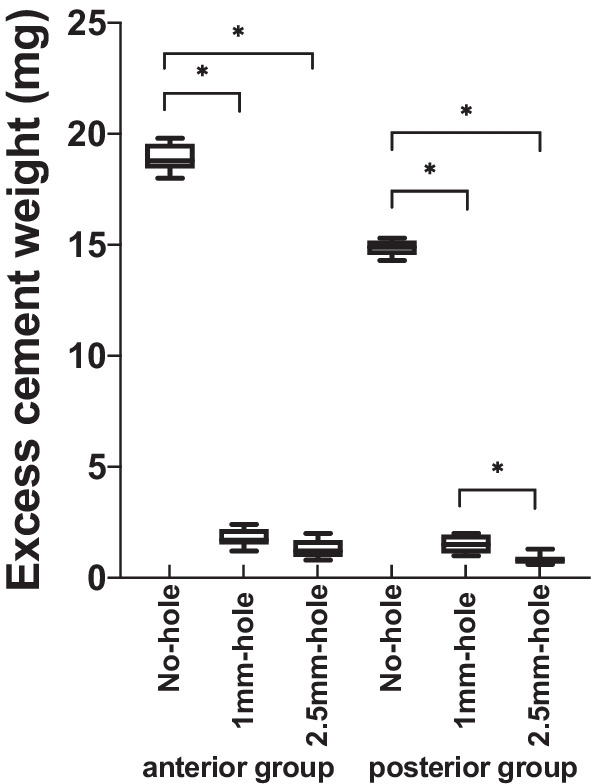
Box-plot chart of weight (ΔW [mg]) of excess cement at the abutment margin for implant crowns with different hole designs (*p < 0.05)

Table 1 and Fig. 4 show the mean and standard deviation for the amount of excess cement at the abutment margin in the anterior and posterior teeth groups. In Group A, the average amount of excess cement was 18.96 ± 0.64 mg, which was the highest for the anterior groups; in Groups B and C, the results were 1.78 ± 0.41 mg and 1.30 ± 0.41 mg, respectively. Levene’s test showed that the variance was equal (p = 0.083). One-way ANOVA indicated significant differences between the anterior teeth groups (F = 3667.949, p < 0.001). The intergroup comparison indicated significant differences between Group A and Groups  B and C (p < 0.001), but no significant difference between Groups B and C (p = 0.053).

**Table 1:** Excess cement weight (ΔW [mg]) of the anterior and posterior teeth groups with different hole designs

					95% confidence interval for mean						
	N	Mean	Std. dev	Std. error	Lower bound	Upper bound	Min	Max	F	P	Intergroup comparison
Group A (ANH)	9	18.96	0.64	0.21	18.47	19.44	19.8	18.0	3667.949		AB: p < 0.001 AC: p < 0.001
Group B (AMH)	9	1.78	0.41	0.14	1.46	2.09	2.4	1.2		p < 0.001	BA: p < 0.001 BC: p = 0.053
Group C (ARH)	9	1.30	0.41	0.14	0.98	1.61	2.0	0.8			CA: p < 0.001 CB: p = 0.053
Group D (PNH)	9	14.87	0.36	0.12	14.59	15.14	15.3	14.3	5043.616		DE: p < 0.001 DF: p < 0.001
Group E (PMH)	9	1.51	0.40	0.13	1.21	1.82	2.0	1.0		p < 0.001	ED: p < 0.001 EF: p < 0.001
Group F (PRH)	9	0.82	0.22	0.07	0.65	0.99	1.3	0.6			FD: p < 0.001 FE: p < 0.001

In the posterior teeth groups, Levene’s test indicated equal variance (p = 0.173), and one-way ANOVA indicated significant differences between groups (F = 5043.616, p < 0.001). Intergroup comparisons showed significant differences between Groups D (14.87 ± 0.36 mg) and E (1.51 ± 0.40 mg) (p < 0.001),  Groups D and F (0.82 ± 0.22 mg) (p < 0.001), and Groups E and F (p < 0.001).

### Retentive strength test

**Fig. 5:**
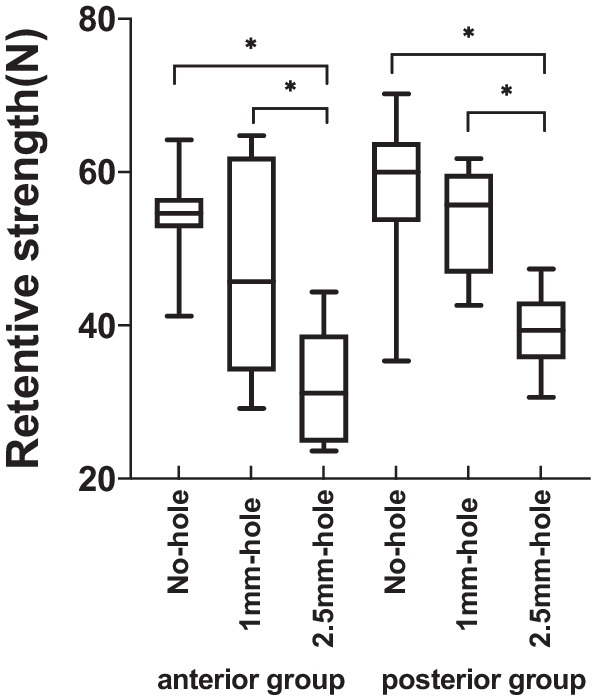
Box-plot chart of retentive strength (N) for implant crowns with different hole designs (*p < 0.05)

Table 2 and Fig. 5 reflect the retentive strength (N) at which the bonded crowns dislocated in the anterior and posterior teeth groups. The mean retentive strengths in anterior teeth groups A, B, and C were 54.16 ± 6.00, 47.63 ± 13.54, and 31.99 ± 7.75 N, respectively; in posterior teeth groups D, E, and F, 57.84 ± 10.19, 53.22 ± 6.98, and 39.48 ± 5.12 N, respectively.

**Table 2:** Retentive strength (N) of the anterior and posterior teeth groups with different hole designs

					95% confidence interval for mean						
	N	Mean	Std. Dev	Std. Error	Lower Bound	Upper Bound	Min	Max	F	P	Intergroup comparison
Group A (ANH)	9	54.16	6.01	2.00	49.54	58.77	64.20	41.20	14.884		AB: p = 0.413 AC: p < 0.001
Group B (AMH)	9	47.63	13.54	4.51	37.23	58.04	64.80	29.20		p < 0.001	BA: p = 0.413 BC: p = 0.026
Group C (ARH)	9	31.99	7.75	2.58	26.03	37.95	44.40	23.60			CA: p < 0.001 CB: p = 0.026
Group D (PNH)	9	57.84	10.19	3.40	50.01	65.68	70.20	35.40	13.786		DE: p = 0.216 DF: p < 0.001
Group E (PMH)	9	53.22	6.98	2.33	47.86	58.59	61.80	42.60		p < 0.001	ED: p = 0.216 EF: p < 0.001
Group F (PRH)	9	39.48	5.1	1.71	35.54	43.41	47.40	30.60			FD: p < 0.001 FE: p < 0.001

In the anterior teeth groups, Levene’s test for the retentive strength indicated that  its variance was not equal (p = 0.013), and Welch’s ANOVA showed significant differences (F = 14.884, p < 0.001). Intergroup comparisons indicated that the dislodging force was the lowest for crowns with a regular 2.5-mm screw access hole (Group C), and significant differences existed between Groups C  and A, (p < 0.001), and Groups C and B, (p = 0.026), but no significant difference was observed between Groups A and B (p = 0.413).

In the posterior teeth groups, the variance  of the retentive strength was equal (p = 0.328), and one-way ANOVA showed significant differences (F = 13.786, p < 0.001). Intergroup comparisons showed that significant differences existed when Group F was compared with Groups D and E (p < 0.001), and that there was no significant difference between Groups D and E (p = 0.126).

## Discussion

Most implant abutments are made of metal, and the edge of the abutment is usually placed approximately 0.5–1 mm below the peri-implant mucosa to avoid unesthetic metallic tints; a major drawback of cement-retained implant restoration is that the residual excess cement underneath the peri-implant mucosa cannot be removed completely, which may lead to peri-implant diseases [11, 13]. This complication may be related to histological characteristics of soft tissue around the implant. Collagen fibers are arranged in parallel or around the neck of the implant, and the soft tissues around the implants are less resistant to mechanical and biological stimulation than those around natural teeth [22–25].

Several techniques have been applied to decrease the amount of cement residue at the abutment margin, including preseating, using polytetrafluoroethylene (PTFE) tape as a cement shield, and hole opening [19–21, 26–29]. Preseating involves the use of an implant abutment analog to fit into the cement-loading crown and extrude the excess cement before the final cementation; one of its shortcomings is that the abutment analog requires customization, which increases expenses [30, 31]. The technique of adapting PTFE tape around the abutment as a cement shield may interfere with the passive seating of the restoration [26]. Compared with other techniques, the hole opening technique does not require extra operating steps, which saves time and expense, making this technique practical and worthy of clinical promotion [17]. In case of problems such as porcelain fracture or screw loosening, implant crowns with holes can easily find the abutment screw and retrieve the abutments and crowns without destruction.

The technique of hole opening in implant crowns has been widely used in clinical practice; however, there is no consensus regarding the appropriate diameter of the hole. According to the manufacturer’s instructions, the diameter of the abutment central screw should be approximately 2–2.3 mm. Therefore, when the diameter of the hole on the crown increases to approximately 2.4–2.6 mm or more, the torque wrench can fasten or loosen the screw directly through the hole. The 1-mm mini venting hole can minimize the damage to the integrity of the crown and have better esthetic results, although the wrench cannot come into direct contact with the screw through the hole.

The results of the current study showed that the presence of a hole on the crown, regardless of its size, could substantially reduce the amount of cement extruded at the abutment margin compared with no-hole crowns, which is consistent with the results of previous studies. For example, Zaugg et al. [19] indicated that venting was the most effective method of reducing excess marginal cement. Jimenez et al. [20] reported that a vent hole on the crown was more advisable than a preseating protocol for improving the performance in terms of reducing excess cement extrusion. In the process of bonding, the air and cement inside the crown would get squeezed out; the only access to excess cement is through the crown margin if no hole exists, and the excess cement may be squeezed deep underneath the peri-implant mucosa. The venting hole on the crown provides a path for cement and air extrusion, and the cement fluid pressure at the margin of the abutment would be reduced when the crown is seated.

In this study, cement extrusion at the margin was reduced by 90.6% and 89.8% with a 1-mm mini venting hole on the crown in the anterior and posterior teeth, respectively; when the hole was the regular screw access hole size (approximately 2.5 mm), it reduced cement extrusion by 93.1% and 94.5% in the anterior and posterior teeth, respectively. Thus, our results indicate that implant crowns with larger holes are more conducive to the discharge of excess bonding cement. However, even a mini-opening can significantly reduce the marginal overflow of the cement. To achieve cost-effective performance and inhibit extrusion of residual excess cement, a smaller hole is clearly superior.

Regarding the retentive strength test, RH crowns had the lowest retention force compared with NH and MH crowns, regardless of whether they were in the anterior or posterior group. Compared with NH crowns, the MH and RH crowns had their retentive strength reduced by 12.1% and 40.9%, respectively, in the anterior teeth, and by 8.0% and 68.3%, respectively, in the posterior teeth. The attenuation of the retention force can be explained by the reduction of the bonding area on the inner surface of the crown. Additionally, the presence of a larger hole on the crown will lead to an increase in the bonding edge line, easier damage of the bonding interface, and more obvious and severe aging of the bonding materials.

The diameter of the hole influences the esthetics and integrity of restoration. Sealing materials, such as light-curing composite resins, are typically used to fill the hole and restore the integrity of the crown [32]. As the diameter of the hole increases, the risk of losing hole-sealing materials may increase. Brandt et al. [33] indicated that laypersons and dentists were able to detect significant esthetic differences in the materials used to fill the holes of dental restorations. The presence of a hole may also compromise the fracture resistance of the restoration: Saboury et al. [34] reported that a central hole with a diameter of 2 mm on implant-supported zirconia restorations decreased fracture resistance. Another study by Du et al. [35] indicated that a full-contour crown with a 1-mm hole should be recommended over holes with diameters of 0-, 2-, 3-, and 4- mm in the posterior teeth region from the aspect of biomechanics by finite element analysis. In contrast, Hussien et al. [36] indicated that screw access channels on implant crowns did not affect the fatigue failure load of implant-supported crowns. In these studies, different materials and experimental strategies were applied, but no consensus on this issue was reached. Based on the evidence available, a larger opening hole on the occlusal surface of the crown may increase the risk of structural weakness, although no clinical consequences could be determined. In the clinical practice of oral implantology, a smaller venting hole can reduce the extrusion of excess cement without decline of retention strength. Together with their esthetic and biomechanical performance, opening holes on implant crowns of approximately 1 mm may be the most efficient, effective, and recommendable.

### Limitations

There were several limitations to this in vitro study. First, we used temporary cement for the cementing test. The bonding materials for implant crowns include temporary or permanent cements. Permanent cements such as glass ionomer and resin cement may make retrieving the crowns difficult; but temporary cements do not form a chemical bond with either the crown or the abutment, making crown retrieval less difficult. Temporary cements become lumpy after solidification, thus the interior surface of the crown is easy to clean when the experiment is repeated. Cement with excellent bonding properties may circumvent the variation in retention strength caused by changes in retention form [37]. Therefore, temporary cement, which provides sufficient bonding strength and allows easy crown retrieval, was used in this study [32, 38–40]. Second, artificial gingiva was not fabricated in this experiment, which enabled easy removal and collection of the extruded excess cement.

Last, we gradually but artificially increased the size of the hole. Prefabrication of a crown with a hole in a dental laboratory may help standardize the size of the hole; however, we required three different crowns (NH, MH, and RH) to complete one set of experiments. The volume of the gap between crowns and abutments, the retention shape, and the inherent friction of different crowns, which are all closely related to the cement spillover and the retention strength experiment, could not be unified. We expect the experimental bias caused by the hole’s hand-opening to be less than that caused by differences between crowns. Because both gradual drilling and prefabricated holes have certain experimental bias, to make a direct comparison and reduce the bias of the cement extrusion and retention strength between different hole designs, we chose the former hole-making scheme.

## Conclusions

In summary, both the amount of excess cement extrusion and the crowns’ retention strength decreased with an increase in the diameter of the venting holes on implant cement-retained crowns. By comparing the implant crowns with different hole designs (no hole, 1-mm hole, 2.5-mm hole), the 1-mm mini venting holes were the most recommended selection based on their balance between reducing cement overflow and preventing deterioration of retention strength.

## Data Availability

Further information on the data set and materials is available from the corresponding author upon reasonable request.

## Abbreviations

ANH: Anterior crowns with no hole
AMH: Anterior crowns with mini venting hole of 1 mm in diameter
ARH: Anterior crowns with regular screw access hole of 2.5 mm in diameter
PNH: Posterior crowns with no hole
PMH: Posterior crowns with mini venting hole of 1 mm in diameter
PRH: Posterior crowns with regular screw access hole of 2.5 mm in diameter
PTFE: Polytetrafluoroethylene
NH: No hole
MH: 1-mm mini venting hole
RH: 2.5-mm regular screw access hole
